# Recent advances in modulating the microbiome

**DOI:** 10.12688/f1000research.20204.1

**Published:** 2020-01-27

**Authors:** Eamonn M.M Quigley, Prianka Gajula

**Affiliations:** 1 Lynda K and David M Underwood Center for Digestive Disorders, Division of Gastroenterology and Hepatology, Houston Methodist Hospital, Houston, Texas, 77030, USA; 2 Department of Medicine, Houston Methodist Hospital, Houston, Texas, 77030, USA

**Keywords:** microbiome, microbiota, antibiotic, probiotic, prebiotic, fecal microbiota transplantation, diet, pharmabiotic

## Abstract

We are in the midst of “the microbiome revolution”—not a day goes by without some new revelation on the potential role of the gut microbiome in some disease or disorder. From an ever-increasing recognition of the many roles of the gut microbiome in health and disease comes the expectation that its modulation could treat or prevent these very same diseases. A variety of interventions could, at least in theory, be employed to alter the composition or functional capacity of the microbiome, ranging from diet to fecal microbiota transplantation (FMT). For some, such as antibiotics, prebiotics, and probiotics, an extensive, albeit far from consistent, literature already exists; for others, such as other dietary supplements and FMT, high-quality clinical studies are still relatively few in number. Not surprisingly, researchers have turned to the microbiome itself as a source for new entities that could be used therapeutically to manipulate the microbiome; for example, some probiotic strains currently in use were sourced from the gastrointestinal tract of healthy humans. From all of the extant studies of interventions targeted at the gut microbiome, a number of important themes have emerged. First, with relatively few exceptions, we are still a long way from a precise definition of the role of the gut microbiome in many of the diseases where a disturbed microbiome has been described—association does not prove causation. Second, while animal models can provide fascinating insights into microbiota–host interactions, they rarely recapitulate the complete human phenotype. Third, studies of several interventions have been difficult to interpret because of variations in study population, test product, and outcome measures, not to mention limitations in study design. The goal of microbiome modulation is a laudable one, but we need to define our targets, refine our interventions, and agree on outcomes.

## Introduction: an overview of the gut microbiome

Strictly speaking, the term “microbiome” refers to the collection of genomes from all micro-organisms in a given environment whereas the term “microbiota” refers to all the micro-organisms found in the environment. In practice, these terms are often used interchangeably. The term microbiota has replaced “flora” in order to emphasize the diversity of microbiota and, in particular, that the human intestinal microbiota normally consists not just of bacteria but also of archaea, viruses, fungi, and multicellular parasites. We now know that it represents a highly evolved and complex ecosystem that plays an important role in the development and maintenance of homeostasis ^1, 2^. Our understanding of the composition and functions of the gut microbiome has been permitted by dramatic and ever-evolving technologies that identify micro-organisms and describe their genetic makeup and metabolism ^3^. We can now annotate, to an ever-increasing depth of detail, what a given microbiome contains, what its constituents are capable of doing (through an interrogation of their genomes), and what they actually produce (employing metabolomics and other techniques). These rapid advances have been facilitated by equally important advances in informatics which allow us to make sense of the enormous databases that microbiota studies generate; techniques such as network analysis and machine learning help to provide meaningful interpretations ^4, 5^. From a variety of laboratory models, such as germ-free and humanized, as well as selective knockout, animal (mostly mouse) models ^6^, insights have been gained into the many interactions between the gut microbiome and the host ^1, 2, 7^. For example, studies on germ-free mice have clearly demonstrated the negative impact of the absence of microbiota on the development and maturation of the immune system ^2, 8, 9^. Now we learn, again from animal models, of the role of the gut microbiome in the development and ongoing functionality of the central nervous system ^10^. Although such studies enable considerable flexibility in terms of manipulation of genotype and phenotype, permit a wide range of possible interventions, and facilitate the collection of various biological samples, they are not without their shortcomings ^11– 13^, and extrapolations to the human condition must be cautious.

What do we know of the human gut microbiome? There certainly has been no shortage of studies on the composition and, to a lesser extent, on the function of the gut microbiome in humans. There are obvious limitations to the scope of studies that can be performed in human subjects in contrast to animal and *in vitro* models, limitations that the reader must be aware of in perusing the literature. It must be remembered that this is a new field and, although progress is being made ^14^, there is still a lack of standardization on many of the technical details of microbiome analysis of human samples ^15^. Although various studies have described links between an altered microbiota and not only gastrointestinal disorders but also diseases as diverse as obesity, diabetes, non-alcoholic fatty liver disease, cancer, and Parkinson’s disease ^16, 17^, these are, at best, associations and do not define causation ^18^. Furthermore, many limitations in patient selection and study design limit the interpretation of many of these studies ^18^.

Although there are some divergent studies ^19^, it is generally agreed that the human gut is relatively sterile at birth ^20– 24^ and acquires its commensal gut microbiome during birth from the mother’s birth canal and thereafter from its oral intake and immediate environment ^25, 26^. Microbial diversity rapidly increases over the first three years of life and then stabilizes at a composition that resembles that of an adult ^25, 26^; this early and critical phase in the development of the microbiome may be especially vulnerable to modulations both beneficial and detrimental ^27^. The origins of diseases that become manifest in adulthood may well be found in the infant microbiome.

Although it is possible that a host of factors influence the adult microbiome, age, geography, diet, and medications have emerged as the principal drivers of inter-individual variation ^28– 36^. However, large population studies revealed that only a small proportion of the variation in the microbiome between individuals could be explained by these and other identifiable factors ^32, 33^—we have much to learn.

Needless to say, there has, of late, been considerable interest in strategies that modulate the gut microbiota as well as in microbiota as sources of novel biologically active molecules ^37– 39^ and predictors of response to various interventions ^40, 41^. Our focus will be on the former: an exploration of strategies to modulate the microbiome. Here, we will consider the range of currently available approaches ( Table 1), explore their impacts, and assess the potential for novel interventions.

**Table 1.** Range of interventions that may modulate the microbiome.

• Lifestyle modification 1. Nutritional intervention and modification; the importance of diet in the short and the long term 2. Caloric restriction 3. Exercise 4. Other lifestyle factors • Clinical interventions 1. Fecal microbiota transfer 2. Antibiotics 3. Prebiotic and probiotics 4. Pharmabiotics 5. Impact of non-antibiotic drugs on the microbiome

## Modulating the microbiome

In considering any intervention that seeks to successfully and, we assume, beneficially modulate the microbiome, one needs to be ever mindful of the complex and dynamic milieu (discussed in the Introduction) which awaits. Simplistic concepts of how a given supplement or medication might influence the microbiota–host interface have generated much hype and even more disappointment. An appreciation of the range of possible interactions between an intervention and host diet, genome, immune system as well as with resident commensals should alert one to the challenges that lie ahead.

We limit the term “modulation” to refer to the manipulation of one or more of the following targets: first, the relative distribution of bacterial species or strains; second, the actual number of bacteria; third, their metabolic activity; fourth, their interactions with the host. There may well be other targets that could be modified—virulence, bacterial antigens, and biofilms, for example—but we have chosen to limit our scope to the aforementioned. In theory, the goal of modulation could be to restore a disrupted or depleted microbiota or transform the existing status quo to induce a “healthier” bacterial community. It must be emphasized that these goals are for now overly simplistic and, despite the claims of various commercial entities that offer microbiome analysis, we are still some way from fully understanding what constitutes a healthy microbiota throughout the gastrointestinal tract. When the microbiome is manipulated, attention must always be paid to the potential for negative outcomes such as the inadvertent introduction or promotion of pathogenic species, transference of antibiotic resistance, or induction of deleterious host responses.

## Lifestyle modification

### Diet and the microbiome

It is now abundantly evident that diet is a major modifier, both in the short and in the long term, of the gut microbiome; this makes absolute sense as, for the most part, microbiota depend for their sustenance on what we ingest. Evidence for the long-term effects of diet comes from studies comparing communities ^28^ or individuals ^29– 33, 42– 44^ with very different dietary habits. These differences reflect lifelong or, at the very least, very long-term dietary practices. In the shorter term, very significant changes in diet, such as reducing fiber intake ^45, 46^, excluding gluten ^47, 48^ or fermentable oligo-, di-, or mono-saccharides and polyols (FODMAPs) ^49^, or dramatically increasing protein intake ^50^, can also impact microbiome composition.

Other dietary components have also been shown to influence microbiota composition ^42^. High-carbohydrate diets promote the growth of *Clostridium* cluster XVIII, *Lachnospiraceae*, ** and *Ruminococcaceae* at the expense of *Bacteroides*, *Bifidobacteria*, and *Enterobacteriaceae*, whereas diets high in fat promote bile-tolerant genera such as *Alistipes*, *Bacteroides*, and *Bilophila* and high-protein diets favor butyrate-producing bacteria such as *Roseburia*, *Eubacterium rectale*, *Faecalbacterium prausnitzii*, *Lactobacilli*, and *Bacteroides* ^42– 44^.

The role of dietary fiber in the development and sustenance of the colonic microbiota has been recognized for decades. Effects of dietary fiber on colonic transit have been linked with the preventative effects of fiber in relation to a variety of diseases as well as in the treatment of disorders such as chronic constipation ^35^. In turn, these beneficial effects may be related to interactions between fiber and colonic bacteria.

Accumulating evidence indicates that effects of fiber on microbiota may be more complex and this should come as no surprise given the heterogeneity of the molecular structures that are found under the umbrella of the term “fiber” ^51^. For example, in the American Gut project, it was found that the number of unique plant species consumed, rather than being a vegan or omnivore, was the best predictor of microbial diversity ^52^. Very specific effects may be linked to the intake of certain fibers – for example, in a randomized clinical trial from China among subjects with type II diabetes mellitus it was found that fibers that promoted the growth of strains that produced short-chain fatty acids resulted a greater amelioration of hemoglobin A1c levels than those that did not ^53^. Clearly, there is much to be learned about the effects of fibers on gut microbiota.

In addition to fiber, dietary ingredients and food additives have been shown to have a substantial impact on the gut microbiota ^44^. Suez and colleagues, for example, found that mice (and, in limited data, humans) consuming non-caloric artificial sweeteners were prone to the development of glucose intolerance, possibly mediated by changes to the intestinal microbiota ^54^. With regard to other supplements and additives, recent research has revealed the role of vitamin D in determining microbiota composition ^55^. Curcumin, which has attracted much interest of late for potential anti-inflammatory and anti-cancer properties, also appears to exert anti-bacterial effects. These include the inhibition of biofilm production and the down-regulation of quorum-sensing virulence factors such as alginate, swarming, and motility ^56– 58^.

It stands to reason that, though less studied, dietary strategies that involve the exclusion of individual but commonly consumed food items or even whole food groups are likely to alter the composition of microbiota. Some of these approaches may, at least in theory, pose problems for the microbiota; the exclusion of FODMAPs, gluten, and fiber, for example, has the potential to deprive important members of the colonic microbiome, such as *Bifidobacteria*, *Prevotella*, and *Bacteroides*, of key nutritional factors such as oligosaccharides and fiber. Although such effects have been demonstrated in the short term ^49^, the longer-term implications are unknown. Changes in the fecal microbiome have indeed been described in relation to this diet; a reduction in *Bifidobacteria* being most notable ^59– 61^. The clinical impact of these and other dietary changes in the long term, in particular, remains unclear ^62^. The Mediterranean diet, for example, has been much lauded for its potential to reduce risk for cardiovascular disease and colon cancer; yet, when formally tested, it did not impact on one microbial metabolite, trimethylamine N-oxide (TMAO), that has been linked with risks for both atherosclerosis and colon cancer ^63^.

What is abundantly clear from all of the above observations is that the impact of diet must be accounted and corrected for in any study of the microbiome in humans. It is also evident that the microbiome contains considerable functional redundancy which allows it to maintain stability in the face of dietary shifts ^64^; this was exemplified by the work of Reichardt and colleagues on short-chain fatty acid production ^65^.

### Caloric restriction

The challenges that dietary studies face are illustrated by an extreme dietary strategy: fasting. Although changes in microbiota diversity and composition have been described in anorexia nervosa and related eating disorders, it has proven difficult to disentangle cause from effect. It would be surprising if fasting, if prolonged, did not impact the gut microbiome ^66^; what remains to be defined is whether there are microbiota signatures specific for eating disorders that might play a role in the pathogenesis of these disorders ^67– 69^. Given the interest that surrounds the potential role of the gut microbiome in obesity, the participation of the microbiome in various calorie-reducing strategies has been the subject of some study. Fasting-induced changes in the microbiota have not only been associated with beneficial metabolic effects ^70^ but also have demonstrated positive effects on intestinal inflammation ^71^ and even central nervous system disorders ^72, 73^. The possible contribution of the microbiome to weight loss and the beneficial metabolic impacts of bariatric surgery have also been explored. A variety of changes in the fecal microbiome have been demonstrated following gastric bypass and other bariatric procedures and were summarized in a recent systematic review ^74^. Guo and colleagues concluded, on the basis of 12 animal experiments and nine clinical studies, that four phyla— *Bacteroidetes*, *Fusobacteria*, *Verrucomicrobia*, and *Proteobacteria*—increased following bariatric surgery but that *Firmicutes*, *Clostridiales*, *Clostridiaceae*, *Blautia*, and *Dorea* were reduced ^74^.

One potentially detrimental consequence of a limited or inadequate dietary intake is that bacteria may turn to host glycans in the mucus layer as substitutes for dietary glycans, thereby upsetting the integrity of the mucus layer which is maintained, in health, through specific bacteria–nutrient interactions ^75, 76^. This disruption of the mucus layer seems to be especially likely to occur in fiber-deprived diets and may render the host more susceptible to pathogens ^77^. This is not to say that the degradation of host glycans is inevitably deleterious, as exemplified by the associations between *Akkermansia muciniphilia* and positive health status ^78^.

### Exercise

Similar challenges are found in attempting to assess the impact of exercise on the microbiome given the almost universal linkage between physical exercise and dietary habit as part of what is referred to as a “healthy lifestyle” ^79^. At one extreme, professional athletes commonly consume much higher amounts of protein which also impact on the composition of the microbiome ^50^. Other interactions also complicate the effects of exercise; for example, body habitus (lean versus obese) significantly affected the impact of 6 weeks of endurance exercise on microbial diversity and short-chain fatty acid concentration ^80^; this finding may reflect diet-related changes in the pre-exercise microbiota. Nevertheless, accumulating evidence indicates that exercise has an independent effect on the microbiome ^81^. For example, an increase in members of the genus *Veillonella* has been identified among marathon runners, and inoculation of these same bacterial taxa into mice was shown to promote endurance by converting exercise-induced lactate into propionate ^82^.

### Other lifestyle factors

Other lifestyle factors, such as cigarette smoking ^83– 85^, alcohol consumption ^85, 86^, and recreational drug use ^87^, have also been linked to changes in the microbiota. With respect to the first of these, the oral microbiota has been of special interest ^88^ given the known relationships between cigarette smoking and oral cancer.

## Clinical interventions

### Fecal microbiota transfer

It has only been in the last decade or so that fecal microbiota transplantation (FMT), though apparently employed on an empiric basic for centuries (if not millennia), has achieved some degree of scientific respectability. Most impressive have been results in recurrent *Clostridioides difficile*–associated disease (CDAD), where cure rates up to and in excess of 90% have been reported ^89^. Various preparations and delivery protocols have been employed with some differences in apparent efficacy ^90^; what remains to be determined is what components of the transplanted fecal microbiota are truly essential for efficacy. FMT has been widely used on an empiric basis in a host of other indications, and instructions for the performance of FMT at home can even be found on the internet. This practice is ill advised: recent reports of severe systemic infections and even death following FMT remind us of the potential hazards of this therapy ^91^. It is notable that results from the use of FMT in other indications are far less impressive than those reported in CDAD. This should come as no surprise as one has now strayed from a disorder caused by a single organism to ones of varying phenotype where, despite considerable efforts, the precise role of the microbiome in etiology remains unclear. Thus, although systematic reviews and some individual trials suggest efficacy for FMT in ulcerative colitis ^92– 94^ and irritable bowel syndrome (IBS) ^95, 96^, results from individual studies provide a far from clear-cut picture; some report either no benefit or even inferior outcomes for FMT ^97– 99^. FMT is clearly a powerful tool but a very blunt instrument; it is a technology in need of considerable refinement once one strays from CDAD. Results in more complex polygenic diseases will undoubtedly require a much more tailored and personalized approach which ultimately should involve the definition of the microbial cocktail that is most effective for each phenotype ^100^. The small intestinal microbiome has long been recognized to play a pivotal role in the pathogenesis of the symptomatology of hepatic encephalopathy; limited clinical trial data suggest that FMT may also have a role here ^101^. The microbiome may play a more fundamental role in the etiology of non-alcoholic fatty liver disease (NAFLD) and its more advanced manifestation, non-alcoholic steatohepatitis ^102^; here, microbiome modulation, including FMT, holds promise ^103, 104^; clinical trials are awaited. It must also be remembered that the gut microbiome includes organisms other than bacteria, such as viruses ^105^, which therefore may be transmitted via or influenced by FMT ^106, 107^.

FMT is not without risk. Not only can infectious agents be transmitted (as illustrated by recent instances of transmittal of extended-spectrum beta-lactamase–producing *Escherichia coli* which proved fatal in one instance) ^108^ but it is also theoretically possible that the transfer of microbial signatures linked to disease states leads to the future emergence of these disorders in the recipient, hence the call for greater regulation of FMT ^109^.

Another barrier to progress is our lack of understanding of how exactly FMT works or how it might work in different clinical situations ^110^. Clues are beginning to emerge, especially in relation to efficacy in CDAD ^111, 112^, but the exact bacterial recipe required for benefits in CDAD or other potential indications has yet to be defined. It seems likely that the composition of the donated material will differ substantially between disease states.

### Antibiotics

A detailed discussion of the indications for and efficacy of antibiotics in human health is beyond the scope of this review. Inevitably, antibiotics that are given orally or that undergo biliary excretion and enterohepatic circulation, regardless of the route of administration, will impact on the gut microbiome to a greater or lesser extent. These “innocent bystander” effects may impair host resistance to pathogens, setting the stage for CDAD or fungal overgrowth, and certain populations are especially at risk ^113^. Antibiotic resistance is a global public health issue; global trends in resistance to one common pathogen, *Helicobacter pylori*, are described as “alarming” ^114^. The human gut microbiota has been described as “a reservoir of antibiotic resistance genes” ^115^; in one study 1,093 antibiotic resistance genes were identified among apparently healthy Chinese individuals ^115^; the potential for horizontal and vertical transfer of such resistance is a source of great concern.

Antibiotics mediate other actions through effects on the microbiota, including effects on inflammation, metabolism, and tumorigenesis; the net impact, whether detrimental or beneficial, is determined by antibiotic, microbial, and host factors ^116– 122^. The long-term implications for human health of these antibiotic effects are only now being appreciated ^122, 123^. Infants seem to be especially vulnerable; accumulating evidence indicates that early and repeated exposure to antibiotics in infancy, even in the very small doses that we ingest through the food chain as a consequence of their use in animal husbandry, may predispose to the development of inflammatory and metabolic diseases in later life ^121– 128^. A call to arms to address the global use of antibiotics is certainly appropriate ^129^.

### Probiotics and prebiotics

The role of prebiotics and probiotics in gastrointestinal health and disease has been the subject of a recent review by one of the authors of this review ^130^ and has also been the subject of a very recent review in this journal ^131^ and therefore will not be repeated in detail here. The International Scientific Association for Probiotics and Prebiotics defines a prebiotic as “a substrate that is selectively utilized by host microorganisms conferring health benefit” ^132^. Probiotics are most commonly defined as “live microorganisms which when administered in adequate amounts confer a health benefit on the host” ^133^. Therefore, in their simplest terms, prebiotics are substances that act as substrates for bacterial digestion and thus proliferation and lead to the generation of metabolites, such as short-chain fatty acids, that are beneficial to the host ^134^, whereas probiotics are live organisms that engage in beneficial interactions with the host.

Substances with prebiotic effects may be found in cereals as well as in plants such as onions, garlic, bananas, chicory root, and Jerusalem artichokes but typically are present at low levels and may not exert prebiotic effects in these forms ^135^. Fructo-oligosaccharides (FOSs) are known to be present in about 36,000 varieties of plants, and wheat is a major source of fructans. More biologically active and selective prebiotics include galacto-oligosaccharides (GOSs), FOSs, oligofructose (OF), chicory fiber, and inulin. Human milk oligosaccharides are important prebiotics provided in breast milk to infants and promote the proliferation of *Bifidobacteria* and, in this manner, have been linked to a number of health benefits ^136^. Other components of breast milk also exert beneficial impacts on the infant’s microbiome and immune system ^137, 138^. Research on prebiotics currently includes a focus on the development of highly selective “designer” prebiotics targeted to impact on specific taxa within gut microbiota; others question the wisdom of the importance or, indeed, the feasibility of the selectivity approach given the functional redundancy that is inherent to the gut microbiome ^139, 140^.

Of late, clinical studies on prebiotics have a strong emphasis on metabolic outcomes such as blood glucose regulation, calcium homeostasis, and weight loss ^132, 135, 141^. Immunological (for example, enhancing antibody responses to vaccines, promoting anti-inflammatory cytokine profiles) ^135, 142^, neurological (some benefits in terms of mood and cognition) ^134^, cardiovascular (improvement in lipid profiles) ^135^, and gastrointestinal effects have also been studied; notable examples are effects on colon transit and benefits in IBS ^61^.

Probiotics have been lauded for centuries for a host of beneficial effects; most await confirmation in high-quality clinical trials ^130, 131^. Nevertheless, a considerable volume of basic science research attests to the ability of various probiotic strains to engage with the mucosal immune system, modulate host metabolism, and even influence gut neuromuscular function ^130, 143^. More remote effects on the liver and central nervous system have also been demonstrated for orally ingested probiotics ^144, 145^. We now have a considerable understanding of how probiotics interact with the host to generate these effects; for example, the molecular basis of the anti-inflammatory effects of certain species of *Bifidobacteria* have been described in great detail in elegant *in vitro* and animal studies ^146^.

As ever, the situation in humans is less straightforward and recent studies emphasize the complexity of the interactions between the administered probiotic, commensal microbiota, and the host that determine the ability of the probiotic to gain a foothold in the gut, colonize, and exert its effects ^147, 148^. A vision of a probiotic as simply displacing “bad” bacteria is clearly very naïve. ****

Of the myriad clinical claims that have been made for probiotics, a few pass muster and have been detailed in several recent reviews and meta-analyses ^130, 131^. The most consistent benefits have been described in the prevention or treatment (or both) of diarrhea in children ^149, 150^, antibiotic-associated diarrhea ^151, 152^, necrotizing enterocolitis ^153^, IBS ^154^, and some phenotypes of inflammatory bowel disease ^155– 157^. It must be stressed that these are aggregate results; though stating that probiotics, in general, have an effect, conclusions are unable to provide direction to the clinician on a specific preparation for a given indication ^157^. Results from individual studies are inconsistent, and major deficits in study design often limit interpretability. Given the tremendous inter-individual variability in the composition of the gut microbiome, it may be unrealistic to expect consistent results from a given microbial strategy in any disease state. Efforts to define what microbial or host factors determine responses could pave the way toward “personalized bacteriotherapy”. There is much to be done.

Probiotics and probiotics may also be combined as synbiotics; although this concept is attractive in theory and synbiotic preparations have enjoyed some notable successes ^158^, synergy is not inevitable ^159^ nor is it always possible to tease out the relative contributions of probiotic or prebiotic to any observed benefit.

### Pharmabiotics

The term “pharmabiotic” has been coined to encompass any material with potential health benefit that can be mined from microbiota, microbiota–host, or microbiota–dietary interactions in the gut ^160, 161^; therefore, it includes not just live organisms but dead or altered organisms as well as bacterial products or metabolites. Some concrete examples include bacterially produced natural antibiotics, bacteriocins ^162^, genetically modified organisms ^163, 164^, bacteriophages ^165, 166^, and short-chain fatty acids ^167^. *E. coli* has been engineered to exert a variety of effects, including overproducing AI-2 signaling molecules and thereby beneficially tilting the Firmicutes/Bacteroidetes ratio in a mouse model of streptomycin-induced dysbiosis ^168^. Vaccination against *Vibrio cholerae* infection in the gut has been achieved with *E. coli* overexpressing both AI-2 and the genus-specific autoinducer-1, CA-1 ^169^; in another example, an engineered *E. coli* seeks and kills *Pseudomonas aeruginosa* via quorum sensing and expression of antimicrobial peptides ^170^. In another approach, the exonuclease Cas3—clustered regularly interspaced short palindromic repeats (CRISPR)-associated protein 3—from type I systems was engineered into a probiotic to selectively and efficiently kill pathogenic bacteria with specific genetic properties ^171^.

Though still rather new in terms of clinical application, these and other technologies offer exciting possibilities for microbiota modulation in the future and may be vital to the resolution of the antibiotic crisis that we currently face. Evolving approaches such as CRISPR-based technologies have revolutionized genome editing and have already been applied to the development of novel antimicrobial strategies ^172– 174^.

### Impact of non-antibiotic drugs on the microbiome

Interventions that modulate intrinsic defense mechanisms against bacterial colonization can be predicted to alter microbiota composition. Acid suppression induced by proton pump inhibitors (PPIs) has been variably but not consistently linked to a predisposition to *Clostridioides difficile* infection, enteric infections, and small intestinal bacterial overgrowth ^175, 176^. Studies of human feces have indeed demonstrated a decrease in *Clostridiales* and an increase in *Actinomycetales*, *Micrococcaceae*, and *Streptococcaceae* among PPI users; these changes were previously associated with an increased susceptibility to this feared complication of antibiotic use ^177– 179^. Similarly, drugs that alter motility and intestinal transit, of which there are many, may also alter microbiota composition ^180^. It is likely that many other drugs engage with the microbiota with resultant enhancement or reduction in efficacy or induction of side effects ^177, 181^, yet another fertile field for future microbiome research.

## Conclusions

The study of the human gut microbiome has emerged as one of the hottest areas of biology and biomedicine and continues to yield tantalizing insights into the contributions of our microbial fellow travelers to health and disease. Accordingly, the modulation of the microbiome to prevent or treat disease has attracted considerable attention, and various strategies have emerged. In most instances, however, progress has been hampered by a lack of clarity on the precise role of the microbiome in a given disorder, variations in human disease phenotype, and variability in formulation and delivery of putative therapies. Progress on all fronts is required to move microbiota modulation to the forefront of medical practice.

## Abbreviations

CDAD, *Clostridioides difficile*–associated disease; CRISPR, clustered regularly interspaced short palindromic repeats; FMT, fecal microbiota transplantation; FODMAPs, fermentable oligo-, di-, or mono-saccharides and polyols; FOS, fructo-oligosaccharide; IBS, irritable bowel syndrome; PPI, proton pump inhibitor
